# Effect of an antepartum Pap smear on the coverage of a cervical cancer screening programme: a population-based prospective study

**DOI:** 10.1186/1472-6963-7-10

**Published:** 2007-01-23

**Authors:** Mari Nygård, Anne-Kjersti Daltveit, Steinar Ø Thoresen, Jan F Nygård

**Affiliations:** 1 Department of Screening-based Research, The Cancer Registry of Norway, Oslo, Norway; 2 Department of Public Health and Primary Health Care, Section for Epidemiology and Medical Statistics, University of Bergen, Bergen, Norway; 3 Medical Birth Registry of Norway, Norwegian Institute of Public Health, Bergen, Norway

## Abstract

**Background:**

Almost one-third of Norwegian women aged 25–69 years invited to have a Pap smear do not attend during the recommended period, and thus constitute a population with high-risk of cervical cancer (CC). Since the incidence of precancerous lesions of the cervix peak with occurrence of pregnancies within the same decade in women aged 25 to 35 years of age, antepartum care presents an opportunity to offer a Pap smear thereby increasing the coverage of the programme. The study objective was to describe the effect of the antepartum Pap smear on the coverage of a cytological CC screening programme.

**Methods:**

Among 2 175 762 women resident in Norway in 31.12.1996, all women who gave birth in 1996–7 were identified from the Medical Birth Registry of Norway. Attendance to the cervical cancer screening was assessed by linkage to the Cytology Registry separately for the pregnant and non-pregnant women cohorts. The results were stratified by age, history of previous Pap smear and history of invitation to the CC screening programme. Logistic regression was used to estimate the relative probabilities of having a Pap smear adjusted for age, screening history, and time since invitation, for pregnant and non-pregnant women, respectively.

**Results:**

69% of the pregnant women had a Pap smear during one year of follow-up since beginning of the pregnancy with the majority taken during the antepartum period. Irrespectively of age or history of having a Pap smear, pregnant women were 4.3 times more likely to have a Pap smear during follow-up compared to non-pregnant women. 63.2% of the pregnant women had a smear as response to the invitation letter compared to 28.7% of the non-pregnant women, OR = 2.1 (95% CI 1.9 to 2.4). As an indication of "over-screening", 5397 pregnant women (57.8%) with a smear shortly before the start of follow-up also had a new Papsmear, compared to 83 023 (32.3%) in non-pregnant.

**Conclusion:**

Pap smear screening during pregnancy increases the coverage of the CC screening programme. The contribution of the antepartum Pap smear to "over-screening" exists but its effect is modest in countries where women on average become pregnant after the start of recommended age of screening.

## Background

Early detection of cervical cancer (CC) has reduced the mortality and morbidity of cervical cancer worldwide [1], and it has been reported that both organised and opportunistic Pap smear taking has lowered incidence rates of CC [2]. However, the importance of obtaining and maintaining a high coverage within the target population has been unanimously recognised [3-8] and several studies support the observation that the decrease in incidence rates is more evident in countries with organised screening programmes [5,9-11].

The Norwegian co-ordinated CC screening programme was introduced in 1995 and 71% of the women 25–69 years of age in Norway had a Pap smear in 1998 to 2000 [12]. More than 50% of the CC cases diagnosed however among the remaining group of non-participants, constituting a population at high-risk of cervical cancer [13].

To minimize the costs of early detection of CC for society, only women without a normal Pap smear in a three years period are identified from the registry files and are invited to be screened [12]. Not all women attend following the invitation; in a Swedish study it was reported that non-attendance to cervical screening was positively associated with time-consuming and economical barriers [14].

The peak age of incidence of pre-cancerous lesions of the cervix peaks with the occurrence of pregnancies in the age range 25–35 [15,16]. From this perspective, antepartum care presents an opportunity to offer a Pap smear to women who otherwise might not go for routine health check-ups, and a means to increase coverage of the programme. Screening of this population can however cause "over-screening" given many of these women might have had a Pap shortly before the start of the pregnancy. Understanding the potential differences in the actual pattern of Pap smear taking activity among pregnant and non-pregnant women would help public health policy makers to recognise conflicting attitudes towards screening and to evaluate the effect of antepartum Pap smear as a routine activity.

The objective of this study is to describe the pattern of Pap smear taking activity and to estimate the impact of Pap smear screening in pregnant women on the overall coverage of the cervical cancer screening programme.

## Methods

### Subjects

All women resident in Norway in 31.12.1996 were identified from the Population Register. Women born before 1900, diagnosed with invasive cervical cancer or underwent hysterectomy before 1996, died in 1997, or had inadequate data for estimating the pregnancy duration were excluded (N = 39 707). The final study population consisted of 2175762 females and the personal identification number (PIN), a unique 11 digit code, identified the subjects.

#### The Norwegian co-ordinated CC screening programme

A thorough description of the CC Screening programme is provided elsewhere [12]. Briefly, the Screening programme in Norway is built around the Cytology Register, which was established in 1992 to administrate the CC screening programme in Norway. It is mandatory to register PIN, date and result of every single Pap smear diagnosed in the country, irrespective of age of the woman, the nature of the health service (private/public) or the indication of the Pap smear. The PIN is used as a key for linkage between the Population and the Cytology Registry in order to identify all 25–69 year old women without a normal Pap smear during a period of three years, which is the recommended screening interval in Norway. From 1995 every women without a Pap smear in the period was identified on a monthly basis and they received personal invitations to participate. In this way, the opportunistic screening was integrated into the organised programme. To date information on more than six million Pap smears is available from the register.

#### The Medical Birth Registry of Norway

Established in 1967, the Medical Birth Registry of Norway (MBRN) was organised to conduct epidemiological surveillance of birth defects and other perinatal health problems in Norway aiming at prevention, as well as health services related to pregnancy, childbirth and the neonatal period, aiming at quality assurance. MBRN routinely registers the PIN and demographic information on the mother and the father, mother's health before and during pregnancy, including chronic diseases, complications during pregnancy and delivery as well as information on the infant, including birth defects and other perinatal problems.

#### Linkage between study population, MBRN and the Cytology Register

The PIN was used to gather information about pregnancies from the MBRN and previous Pap smears from the population-based Cytology Register for each subject.

From the MBRN records all 116 810 women who gave birth (or had abortion after the 16th gestation week) in 1996–7 in Norway (N = 121 400) were identified with information about exact date of birth, duration of the pregnancy, details about pregnancy outcome, multiple offspring, birth weight, and information on previous pregnancies. When there was more than one pregnancy registered per women for the period 1996–7 (2615 women had two and four women had three consecutive records during two years period) the first was included. When there was more than one offspring per birth (1904 twins, 58 triples and 2 women had 4 offspring) we used information about the first born. For 105 818, women the date of the last menstruation was available and used as the beginning of the antepartum period. For 10 992 women (9.4%) this information was missing and beginning of the pregnancy period was estimated by subtracting from date of birth 41 weeks if birth weight was > 3400 gram, 40 weeks if 3200–3400 gram, 39 weeks if 2800–3000 gram, 38 weeks if 2600–2800 gram, 37 weeks if 2400–2600 gram, 36 weeks if 2200–2400 gram, 35 weeks if 2000–2200 gram, 34 weeks if 1800–2000 gram, 33 weeks if 1600–1800 gram, 32 weeks if 1400–1600 gram, 31 weeks if 1200–1400 gram, 30 weeks if 1000–1200 gram, 29 weeks if 800–1000 gram, 28 weeks if 600–800 gram, 27 weeks if 400–600 gram.

For each study subject we identified the date and the diagnoses of the Pap smears from the Cytology Register. Subjects who received an invitation letter from the Cancer Registry of Norway were identified, together with the posting date of the invitation letter.

### Study cohorts

We categorised the following three mutually exclusive cohorts, within the study population:

1. *Pregnant women cohort (C_*preg*_)*: subjects giving birth during the period ± 3 month from the date 31.12.1996. T_0 _is the date denoting the start of the follow-up. For subjects within the pregnant women cohort, T_0 _was the date when the antepartum period started, estimated individually for each subject as described above.

2. *Reference cohort (C_*ref*_)*: subjects who neither gave birth nor were pregnant during this calendar period respective to the follow-up period of the pregnant women e.g. from 01.01.1996 to 31.12.1997. For the Reference cohort, T_0_, the date marking the start of the follow-up was randomly chosen for each subject from the same calendar period as for the C_*preg*_.

3. *Mixed cohort*: all other subjects in the study population who gave birth in 1996–7 but were not included in the C_*preg*_.

Pregnant women and reference cohort were followed up for one year (since T_0_) in the Cytology Register for Pap smears. To identify the pattern of attendance to screening for each subject, time since last Pap smear before T_0 _was identified and stratified as the last Pap smear taken 1, 2, 3 or > 3 years earlier. To evaluate the response to the invitation letter, we identified subjects who received an invitation to screening and stratified analyses by the assessed time since the invitation was mailed in relation to T_0_, i.e. 24 to 2 months prior to T_0_, one month prior to 3 months after the T_0_, more than three months after the T_0_.

### Statistical analyses

The Kaplan-Meier method was used to estimate the cumulative probability of having a Pap smear since T_0_.[17]

We used unconditional logistic regression to compare the odds of having a Pap smear within 12 month after inclusion in the study for *C*_*ref *_and *C*_*preg*_. Odds ratios (OR) with 95% confidence intervals (95% CI) were estimated both crude and adjusted for age, screening history and time since invitation.

## Results

Altogether, 24 297 women were assigned to the *C*_*preg*_, 2 060 118 to the *C*_*ref *_and 91 347 to the mixed cohort. For the three cohorts, age distribution, history of the Pap smear and history of the invitation are given in Table 1. 44% of the *C*_*ref *_had Pap smears during the three years before T_0 _compared to 78 % among the *C*_*preg*_, reflecting the wider age span in the *C*_*ref *_(all female population) compared to *C*_*preg *_or mixed cohort (women of reproductive age). When only women 15 to 49 years of age were analysed, the proportion having had smears in the *C*_*ref *_was 65% (data not shown). The occurrence of a Pap smear within one year after T_0 _was assessed separately for the *C*_*ref *_and *C*_*preg *_(Table 2). Overall, the proportion of women with Pap smears in the *C*_*preg *_was three times higher (68.7%) than the *C*_*ref *_(20.8%).

**Table 1:** Baseline characteristics of the Norwegian female population* in 1996 by pregnancy status, age, history of Pap smear and invitation to the cervical cancer screening programme

	**Pregnancy status**		
	Reference cohort 2 060 118 (%)	Pregnant cohort 24 297 (%)	Mixed cohort 91 347 (%)

**Age-groups**			
< 14	445 928 (21.7)	18 (0.07)	11 (0.01)
15–19	125 039 (6.1)	1688 (7.0)	4090 (4.5)
20–24	130 147 (6.3)	6087 (25.0)	20 103 (22.1)
25–29	127 742 (6.1)	9112 (37.5)	34 809 (38.1)
30–34	131 081 (6.4)	5555 (22.9)	23 530 (25.8)
35–39	144 959 (7.0)	1653 (6.8)	7756 (8.5)
40–44	146 978 (7.1)	178 (0.7)	1024 (1.1)
45–49	149 296 (7.2)	6 (0.02)	24 (0.03)
50–69	394 224 (19.1)	-	-
70+	267 724 (13.0)	-	-
			
**Time since last Pap smear before T**_**0**_			
0–12 months	441 735 (21.4)	9342 (38.4)	36 318 (39.8)
13–24 months	278 490 (13.5)	5934 (24.4)	23 304 (25.5)
25–36 months	181 158 (8.8)	3662 (15.1)	13 113 (14.4)
> 36 months	1 158 735 (56.2)	5359 (22.1)	18 612 (20.4)
			
**Time since invitation**			
No invitation	1 567 788 (76.1)	18 374 (75.6)	75 393 (82.5)
24–2 months prior to T_0_	229 417 (11.1)	2596 (10.7)	989 (1.1)
1 months prior-3 months after T_0_	132 981 (6.5)	2256 (9.3)	3524 (3.9)
> 3 months after T_0_	129 932 (6.3)	1071 (4.4)	11 441 (12.5)

* 39 707 were excluded because they were either born before 1900, diagnosed with invasive cervical cancer and/or underwent hysterectomy before 1996, died in 1997 or had inadequate data for estimating duration of the pregnancy

**Table 2:** Proportion of women with Pap-smear during one year follow-up by age, history of Pap smear and invitation to the cervical cancer screening programme

	Women with Pap smear(s) in follow-up of 12 months	
	Reference cohort N (%)	Pregnant cohort N (%)
**Age-groups**		
< 14	1449 (0.3)	14 (77.8)
15–19	24 181 (19.3)	1115 (66.1)
20–24	44 720 (34.4)	4261 (70.0)
25–29	45 268 (36.3)	6349 (70.0)
30–34	44 844 (34.2)	3725 (67.1)
35–39	47 182 (32.6)	1097 (66.4)
40–44	48 445 (33.0)	120 (67.4)
45–49	49 775 (33.3)	3 (50.0)
50–69	95 905 (24.3)	-
70+	14 056 (5.2)	-
		
Time since last Pap-smear* before T_**0**_		
0–12 months	83 023 (32.3)	5397 (57.8)
13–24 months	59 021 (36.9)	4289 (72.3)
25–36 months	47 100 (46.0)	2909 (79.5)
> 36 months	65 496 (23.1)	4072 (76.2)
		
Time since invitation*		
No invitation	185 991 (33.2)	12 732 (69.4)
24–2 months prior to T_0_	26 664 (25.0)	1737 (67.0)
1 months prior-3 months after T_0_	26 455 (37.4)	1683 (74.6)
> 3 months after T_0_	15 530 (23.7)	515 (48.1)

* age-groups 15–44 only

**Figure 1:**
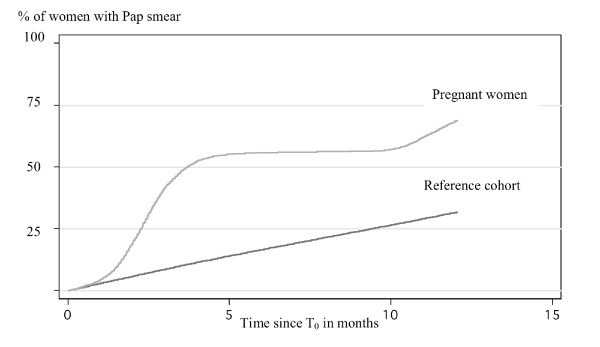
Cumulative probability for Pap smear since T_0 _for women in age of 15–44 years, for the Reference and Pregnant women cohorts by Kaplan Meieranalyses. Y-axes refer to the proportion of women with Pap smear and X -axes refer to time in months from the start of follow-up. The increase of proportion of women with Pap smear during follow-up time is depicted separately for Pregnant women cohort (light grey line) and for the Reference cohort (black line).

Only women 15 to 44 years of age were included in the stratified analyses by Pap smear history, invitations and Kaplan-Meier analyses for estimating cumulative probability for having a Pap smear since T_0_. (Figure 1). By the end of the follow-up of one year, 31.6% of the *C*_*ref *_had a smear compared to 67.8% of the *C*_*preg*. _The linear increase of the cumulative probability of the Pap smear is an expected pattern for the screened population in Norway indicating the appropriate selection of the *C*_*ref *_for current study.

### Compliance with the recommended screening interval of three years

80% of the *C*_*preg *_and 46% of the *C*_*ref *_women with a Pap smear 2–3 years prior to T_0 _and next Pap smear within one year were classified as those who were following the recommendations. 76.2% of the *C*_*preg *_and 23.1% of the *C*_*ref *_women women with a smear more seldom than recommended screening interval had a Pap smear within one year. 57.8% of *C*_*preg *_and 32.3% of the *C*_*ref *_women with a smear taken shortly before and after T_0_, were classified as "over-screened".

### Attendance to the screening following invitation letter

When invitation was mailed close to T_0_, defined as 4 months, from one month prior to T_0 _to three months after T_0_, 74.6% of *C*_*preg *_had a Pap smear in during one year of follow-up, compared to 37% of the *C*_*ref*_.

Pregnant women were almost 5 times more likely to have a Pap smear within one year of T_0 _compared to the non-pregnant women, crude OR = 4.7 (95% CI 4.6 to 4.8). After adjusting for age, time since last Pap smear and time since invitation, this estimate was somewhat lower, adjusted OR = 4.3 (95% CI 4.2 to 4.4) (Table 3). Women who had the last Pap smear more than 3 years since T_0 _had a smaller probability of a new Pap smear during the next one year compared to women who had a last smear taken within one year of T_0_, irrespectively of the pregnancy status, adjusted OR = 0.76 (95% CI 0.75 to 0.77). When this estimate was limited to *C*_*preg*_, the OR was 2.6 (95% CI 2.4 to 2.8), compared to OR of 0.73 (95% CI 0.72 to 0.74) for the *C*_*ref *_only. Compared to the non-pregnant women, pregnant women were 2.1 times more likely (95% CI 1.9 to 2.4) to have a Pap smear following the invitation letter if the latter was mailed close to the beginning of T_0_, compared to the non-pregnant women.

**Table 3:** Probability for Pap smear within one year since T_0_. Crude and adjusted odds ratios (OR) and 95% confidence intervals (CI) were estimated by logistic regression

	Probability for Pap smear within 12 month since T_0_			
	Crude OR	95% CI	Adjusted OR	95% CI
Reference cohort	1	ref.	1	ref.
Pregnant ccohort	4.72	4.59–4.85	4.28	4.16–4.40
				
**Age-groups**				
15–19			0.53	0.52–0.54
20–24			0.95	0.94–0.97
25–29			1	ref.
30–34			0.91	0.89–0.92
35–39			0.85	0.84–0.87
40–44			0.88	0.87–0.90
				
Time since last Pap-smear*				
0–12 months prior to T_0_			1	ref.
13–24 months prior to T_0_			1.22	1.20–1.24
25–36 months prior to T_0_			1.77	1.75–1.80
> 36 months prior to T_0_			0.76	0.75–0.77
				
Time since invitation*				
No invitation			1	ref.
24–2 months prior to T_0_			0.91	0.89–0.92
1 months prior-3 months after T_0_			2.12	1.89–2.38
> 3 months after T_0_			1.00	0.92–1.09

## Discussion

We found that smear taking activity during pregnancy in Norway was high, with 69% of pregnant women having had a Pap smear during one year of follow-up. Most of the Pap smears from pregnant women were taken during the antepartum period, within 4 months from the start of the pregnancy, and therefore they will be further referred as antepartum Pap smears. Norwegian guidelines state that 1^st ^trimester antepartum Pap is recommended given no normal smear was taken during the period of 2,5 years prior to the visit [16]. This recommendation is given regardless of age because the average 1^st ^full-time pregnant Norwegian woman is 29 years old and should already been participating at the screening. However, 58% of pregnant women with a smear taken shortly before had also an antepartum Pap, reflecting the real-life situation, indicating poor adherence to the guidelines.

### Effect of an antepartum Pap smear to the coverage

Compared to non-pregnant women, pregnant women were 4.3 times more likely to be screened by Pap smear during one year period, irrespectively of their age or screening history. As much as 76% of the pregnant women without a smear in three years prior to start of pregnancy had a smear in follow-up compared to 23% of the non-pregnant women. This large difference can partially be explained by the fact that more women in age of 15–24 years were included into the Reference cohort and therefore, should not have been screened at all. The age issues were taken into account by estimating the risk of a smear (adjusted for pregnancy status, age, or invitation) for women without the smear in three years period and for women who had smear shortly prior to the start of the study. It was somewhat surprising to observe that women without a smear within the last three years were less likely to have a smear compared to women who were screened lately, OR = 0.76. This figure can be explained by the observation that women with frequent smear taking activity were more likely to continue such a pattern, whereas women who had a smear taken rarely or never, were less likely to have a smear in the near future. One can postulate that one of the most important obligations of an organized CC screening programme is to minimise the proportion of women without a smear: and as a consequence, this risk estimate should eventually increase. As an example, pregnant women who had a last Pap smear more than three years prior to the start of the study, were 2.6 times more likely to have a smear in one year compared to women with a smear shortly before start of the study. The comparative figure for the non-pregnant women was 0.73. Together with the fact that pregnant women showed a higher probability of a favourable response to smear taking by invitation letter than non-pregnant women OR = 2.12 (95% CI 1.89 to 2.38) it implies that Pap smear in pregnancy increases the coverage of the programme.

Elucidating as to why almost two-thirds of non-pregnant women aged 15–44 years with an invitation letter did not have Pap smear, and why there was a three times higher attendance rate among pregnant women compared to non-pregnant women is important. Possibly an explanation lies in the relatively high work load of the women: they are usually either studying, have just joined the work force, have established a family, already have small children to tend to, or carry out a combination of these duties. The need for a regular check up for precursors of cervical cancer could be given less priority in such demanding/real-life settings. This explanation is in line with a Swedish study, where Eaker concluded that non-attendance to cervical cancer screening is rather practically rooted.[18] A study from the U.S. identified no effect of either patient or physician reminders on Pap smear completion, while patients with a chronic illness had a three times higher probability of Pap smear completion,[19] indicating that access to health care is a lesser issue for those with chronic diseases.

### Pap smear screening among women before screening age

Only 32% of the pregnant women were < 25 years old and expected to be not screened due to young age. Women aged 15–19 had Pap smears in follow-up period more seldom than women aged 25–29 years, OR = 0.53. However, the probability of a smear was 66.1 % for pregnant women compared to 19.3% for non-pregnant women indicating that antepartum Pap contributed to increased screening among young women. In absolute terms, 1129 pregnant women aged 15 to 19 years had a smear during the one-year period. If all pregnant women in this young age group would have had a smear, the consequent number would have been 1706 compared to 25630 non-pregnant young women. It is clear that other factors than pregnancy seems also relevant in explaining the Pap smear-taking activity among young women. It should be remembered that a sexually transmitted virus, human papilloma virus, is responsible for developing pre-invasive cervical lesions and CC [20,21]. Pregnant women irrespective of age therefore represent a population with a past or current exposure to sexually transmitted infections. However, many mild dysplastic cervical lesions are subjected to regress, as we have shown in our previous study on young women [22], suggesting that mass-screening in young ages is unwarranted.

### Does Antepartum Pap smear contribute to "over-screening"?

Altogether 32% of non-pregnant and 58% of pregnant women were classified as "overscreened", as defined by repeated Pap-smears in short period of time, emphasizing the fact that pregnant women taking an antepartum Pap cannot be solely the reason for the frequent screening observed. Further, the overall proportion of pregnant women in the population is small: expressed in the absolute numbers as much as 83 023 of non-pregnant compared to 5397 pregnant women underlines that the antepartum Pap does not substantially constitute to the overall number of extra smears taken.

In the current study in assessing the effect of pregnancy on coverage only we did not consider the possibility that Pap smears taken shortly before the start of the follow-up were abnormal, and as defined, that they should be followed up soon after with a Pap smear. Nor did we consider that the onset of clinical symptom(s) leading to a new Pap smear. These are relevant concerns that we cannot appropriately address in this study design. However, there are no strong reasons to suspect large difference in the two cohorts in these respects. Taking into consideration that the proportion of abnormal Pap smear is low in the programme: approx. 86% of all the smears are normal, and any existing differences in the distribution of abnormal smears in the pregnant and non-pregnant women are likely to be only weakly affect the estimates of coverage.

It is natural combine the antepartum visits with the distribution of the health education among women and several authors demanding the routine antepartum smear [23,24] in order to improve diagnosis of the CIN. However, the decision on recommending Pap smears for all pregnant women should be based on information on the accuracy of the antepartum Pap to diagnose underlying pre-invasive lesion, the impact on coverage and on the mean ages of pregnancy in given country.

## Conclusion

Several studies have been performed to assess the value of a Pap smear collected during the antepartum and/or postpartum period. Conclusions regarding the necessity of routine Pap smear(s) during pregnancy are often missing or, sometimes contradictory, reflecting that information on availability or history about cervical cancer screening is often unavailable. This registry linkage study has been conducted to estimate the effect of Pap smear during pregnancy on the coverage, and our study suggests that the provision of Pap smears to all pregnant women in Norway will increase the coverage of the programme. The contribution of such an action on over-screening and screening among young women is likely to be modest in the country where in the average, women become pregnant after the recommended age of screening.

## Competing interests

The author(s) declare that they have no competing interests.

## Authors' contributions

MN, AKD, SØT and JFN participated actively in the design of the study and helped to draft the manuscript. JFN carried out the statistical analyses; MN and AKD participated in data base compiling; MN had main responsibility to perform this study. All authors read and approved the final manuscript.

## Pre-publication history

The pre-publication history for this paper can be accessed here:


